# Effectiveness and Safety of Recombinant Zoster Vaccine in Rheumatic Diseases: Real-World Evidence from a Single-Centre Italian Cohort

**DOI:** 10.3390/vaccines13121227

**Published:** 2025-12-05

**Authors:** Silvia Valentini, Jurgen Sota, Irene Fineschi, Edoardo Conticini, Estrella Garcia-Gonzalez, Emilio D’Ignazio, Marco Bardelli, Stefano Gentileschi, Marta Fabbroni, Francesca Bellisai, Paolo Falsetti, Enrico Selvi, Luca Cantarini, Bruno Frediani, Caterina Baldi

**Affiliations:** 1 Rheumatology Unit Department of Medicine, Surgery and Neurosciences, University of Siena, 53100 Siena, Italy; valentini.reumatologia@gmail.com (S.V.); sota.reumatologia@gmail.com (J.S.); estre79@gmail.com (E.G.-G.); enrico.selvi@gmail.com (E.S.);; 2 Usl Sud-Est Tuscany, 52100 Arezzo, Italy

**Keywords:** RZV, Shingrix, herpes zoster, vaccines, systemic autoimmune diseases, immunosuppressive therapy

## Abstract

Background: Patients with rheumatic diseases (RMDs) are at increased risk of herpes zoster (HZ), particularly when receiving immunosuppressive treatment. While recombinant zoster vaccine (RZV) has shown high effectiveness in the general population, evidence in rheumatologic patients remain limited due to their exclusion from pivotal trials. Objectives: To evaluate the effectiveness of RZV and to collect additional safety data in a heterogeneous cohort of rheumatologic patients, compared with a control cohort from the general population. Methods: We conducted a retrospective study including 179 adults who received two intramuscular doses of RZV between January 2021 and June 2025. The cohort included 114 patients with RMDs and 65 individuals from the general population. Effectiveness was defined as the ability to prevent HZ reactivation while safety concerns were recorded as any adverse event temporally associated with the vaccination. Results: We observed a statistically significant reduction in terms of VZV relapses following vaccination (*p* < 0.001). Among patients diagnosed with RMDs, only one case of HZ recurrence was observed 14 weeks after vaccination, with no significant difference compared to general care patients. One patient experienced a disease flare requiring glucocorticosteroids. RZV demonstrated a favourable safety profile, with minor adverse events (fever, injection-site reactions, headache and myalgia) reported in 17.5% of patients after the first dose and 21.5% after the second. No significant association was observed between adverse events and advanced immunosuppressive therapy. Conclusions: RZV displayed an effective and reassuring safety profile in a heterogeneous cohort of patients affected by RMDs, irrespective of the diagnosis and the ongoing therapy. This supports the broader use of RZV as a safe and valuable preventive strategy in patients with RMDs.

## 1. Introduction

Herpes zoster (HZ), also called shingles, results from the reactivation of latent varicella-zoster virus (VZV), which initially causes primary varicella infection (chickenpox) during childhood. Following the primary infection, the virus remains dormant in the sensory dorsal root ganglia and may reactivate later in life, particularly under conditions of age-related immunosenescence or immunosuppression [1]. The main risk factors for the development of herpes zoster include advanced age, a family history of herpes zoster, female sex, psychological stress and immunosuppressive conditions such as HIV/AIDS, malignancies and autoimmune disorders [2]. Age represents the primary risk factor for herpes zoster, particularly among patients in treatment with statins [3]; consequently, current vaccination guidelines are mainly based on the age of individuals. In Europe, recommendations regarding herpes zoster (HZ) vaccination vary across countries, depending on the mean age of the general population and national definitions of individuals at increased risk for HZ. The age cut-off for vaccination eligibility in the general population ranges from 50 years old age (yoa) in some countries, 60 yoa in others, and 65 yoa in others, including Italy [4].

Patients with systemic immune-mediated rheumatic diseases are at increased risk of infections compared to the general population, due to multiple factors including disease activity [5], comorbidities and treatment with immunosuppressive agents, such as glucocorticosteroids and disease modifying anti-rheumatic drugs (DMARDs) [6]. Of note, an increased risk of developing herpes zoster (HZ) has been observed, particularly among patients treated with Janus kinase (JAK) inhibitors [7]. Hence, both the 2019 EULAR update and the 2022 ACR guidelines recommend HZ vaccination in this group of patients [8,9]. In both cases, these recommendations refer to the recombinant subunit vaccine (RZV).

Thus, two vaccines are currently available for the prevention of herpes zoster: adjuvanted recombinant zoster vaccine (RZV) and zoster vaccine live (ZVL). ZVL is a live attenuated vaccine, first approved in 2006 by the Food and Drug Administration (FDA) for adults >50 years old, subsequently expanded to include patients aged over 50 years. Being a live attenuated vaccine, ZVL is contraindicated in immunocompromised population due to the risk of vaccine-derived viral infection.

RZV is a non-live, adjuvanted recombinant subunit (containing the ASO1B adjuvant), approved in 2017 by the Food and Drug Administration for prevention in adults >50 years old and administered in two doses, 2 to 6 months apart [10]. A comparative meta-analysis evaluating the effectiveness and safety of herpes zoster vaccines in the general population showed that the recombinant zoster vaccine (RZV) has a statistically significant effectiveness compared to ZVL in preventing both HZ and post-herpetic neuralgia (PHN), statistically significant higher reactogenicity defined by the incidence of injection-site reactions and systemic reactions, and a safety profile comparable to ZVL [11]. RZV has shown high effectiveness in preventing HZ in the general population. In two phase III trials (ZOE 50–70), RZV demonstrated an effectiveness of 97.2% in adults ≥50 years [12] and 89.8% in those ≥70 years [13]. In a post hoc analysis, effectiveness remained high (90.5%) among participants with at least one immune-mediated disease [14]. Although recent data have provided preliminary evidence on the effectiveness, safety profile, and immunogenicity of RZV patients with rheumatologic diseases [15], evidence from large-scale studies are still lacking and warrants further investigation. On the other hand, existing data on RZV safety profile are limited by the under-representation of specific conditions and by the confounding influence of comorbidities, thereby further limiting the applicability of these findings to current clinical practice [16]. The present study aims to evaluate the effectiveness of the RZV and to provide additional safety data in a heterogeneous cohort of patients with systemic immune-mediated rheumatic diseases, compared to a cohort from the general population.

## 2. Materials and Methods

### 2.1. Study Participants

Medical records from adult patients receiving two intramuscular doses of RVZ vaccine (Shingrix) were retrospectively reviewed. The study cohort was composed by a subgroup of patients diagnosed with a systemic immune-mediated rheumatologic disease and a control group with data retrieved from the general practitioner (GP) databases. Patients included in the rheumatologic cohort were identified among individuals attending the Rheumatology Unit of a tertiary referral centre. The general care group consisted of vaccinated individuals recruited from the outpatient clinic of a general practitioner serving the same catchment area as the hospital. Data from both case and control groups were collected between January 2021 and June 2025, using the date of the first vaccine dose as the baseline time point. For the rheumatologic patient group, demographic and clinical data—including information on the number of herpes zoster (HZ) recurrences and ongoing treatments—were collected through questionnaires administered during clinical visits or via telephone interviews. For the general care group, demographic and clinical information (including comorbidities, number of HZ recurrences, and dates of the two vaccine doses) were obtained from the general practitioners’ database. For both groups, exposure data—including the dates of the two HZ vaccine doses—and related outcomes were obtained through patient recall. Patients’ characteristics were retrieved from clinical records, diagnostic codes, and prescription data. Patients in the rheumatologic cohort met the most up-to-date classification criteria for their respective diagnoses. All participants were educated about the study objectives and provided informed consent to participate.

### 2.2. Aims and Endpoints

The primary aim of the study was to evaluate the effectiveness of the RZV in a heterogeneous cohort of patients with rheumatologic diseases encompassing a broad spectrum of diagnoses. The study secondary aims were the following:

(1) Collect additional safety data on RZV in a large cohort with adequate representation of the main rheumatologic diagnoses and to characterize adverse events reported as adverse events following immunization (AEFI).
(2) Identify potential predictive factors of AEFI among demographic and clinical variables.

The primary aim was assessed via any statistically significant differences in terms of VZV reactivation before and after vaccination, standardized in terms of patient-time (HZ episodes per 100 patient-year).

An AEFI was defined as any undesired medical event temporally associated with vaccination, regardless of causal relationship. The AEFIs occurring from the administration of the first vaccine dose until the data collection phase, conducted via phone interviews in June 2025, were included. Disease relapse was included in the safety profile and defined as a clinically meaningful increase in disease activity, as evidenced by worsening of signs and/or symptoms, with or without corresponding changes in laboratory parameters, requiring medical reassessment and/or therapeutic adjustment.

### 2.3. Statistical Analysis

Data were analyzed using IBM SPSS 25.0 Statistics for Windows (IBM Corp., Armonk, NY, USA). The Shapiro–Wilk test was used to assess normality of continuous variables. Mean and standard deviation (SD) or median and interquartile range (IQR) were employed for quantitative variables as appropriate, while qualitative variables were reported as absolute frequencies and percentages. Group differences were assessed using the Mann–Whitney U test, and correlations between continuous variables were evaluated using Spearman’s rank correlation coefficient. Crude incidence rate was used to calculate events/total person-time, followed by incidence rate ratio with 95% C.I.

Predictors of AEFI were analyzed with binary logistic regression with the enter method. The following variables were selected based on their clinical plausible confounding potential alongside essential demographics: group, number of attacks before first dose of VZV vaccine, immunosuppressive treatment, comorbidities, and treatment with prednisone or equivalent. The threshold of statistical significance was set at a *p*-value of 0.05 and all tests were 2-tailed.

## 3. Results

We enrolled a total of 179 patients (114 patients diagnosed with a systemic rheumatologic disease and data from 65 patients without immune-mediated diseases were collected from GP). The male-to-female ratio was 0.66% and all patients received two doses of RZV.

**Figure 1:**
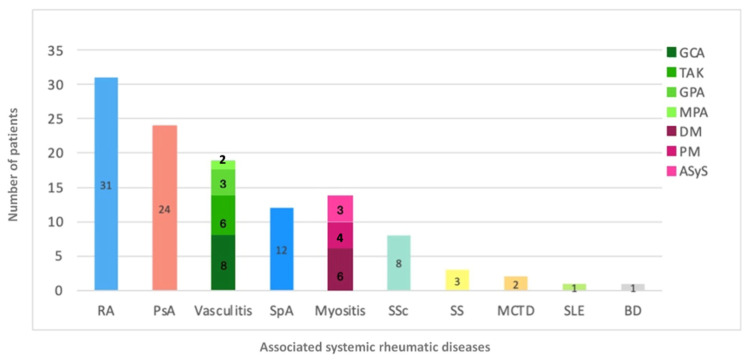
Diagnosis distribution in the rheumatologic subgroup. List of abbreviations: *ASyS* Antisynthetase syndrome, *BD* Behçet disease, *DM* Dermatomyositis, *GCA* Giant cell arteritis, *GPA* Granulomatosis with polyangiitis, *MCTD* Mixed connective tissue disease, *MPA* Microscopic polyangiitis, *PM* Polymyositis, *PsA* Psoriatic arthritis, *RA* Rheumatoid arthritis, *SpA* Spondyloarthritis, *SS* Sjögren syndrome, *SSc* Systemic sclerosis, *SLE* Systemic Lupus Erythematosus, *TAK* Takayasu arteritis.

The mean age of participants at enrolment, defined as the date of the first RZV dose, was 61.0 ± 14.3 years in the rheumatologic group and 77.0 ± 6.7 years in the general population group. Within the rheumatologic cohort, diagnoses were distributed in order of frequency as reported in Figure 1.

Ongoing therapies included monotherapy with biologic DMARDs (bDMARDs) (n = 38, 33.33%), conventional synthetic DMARDs (csDMARDs) (n = 11, 9.6%), JAK inhibitors (n = 31, 27.19%), mycophenolate mofetil (n = 9, 7.89%), and combination therapy of csDMARDs + bDMARDs (n = 16, 14.03%), csDMARDs + JAK inhibitors (n = 8, 7.02%), bDMARDs + JAK inhibitors (n = 2, 1.75%). In the general care group, the main comorbidities included cardiovascular disease (n = 28; 50.90%), hypertension (n = 18, 32.73%), chronic respiratory diseases such as COPD and emphysema (n = 11, 20%), dyslipidaemia (n = 12, 21.82%), cancer (n = 12, 38.20%), benign prostatic hyperplasia (n = 6, 10.9%), and diabetes mellitus (n = 10, 18.18%). A history of HZ prior to the vaccination with RZV was documented in 55 out of 114 patients (48.2%) in the rheumatologic group and in 44 out of 65 individuals (67.7%) in the general population group. Demographic, clinical and therapeutic data for the entire cohort are detailed in Table 1.

A statistically significant reduction was found in terms of VZV relapses before and after vaccine. More in detail, the number of HZ episodes pre-vaccination and post-vaccination with RZV is equal to 9.29 per 100 patient-year and 0.45 per 100 patient-year, respectively, with an IRR of 0.0484 (95% C.I. 0.0024–0.965). Treatment with immunosuppressants overall and JAK-inhibitors considered separately did not impact the mean reduction in RZV relapses (*p* = 0.278 and *p* = 0.635, respectively). Similarly, age was not correlated with the mean reduction in RZV relapses (rho coefficient −0.030, *p* = 0.775). Among the 114 rheumatologic patients, only one case exhibited a recurrence of HZ 14 weeks after the second dose of vaccine. The mean number of recurrences of the rheumatologic disease was negligible, approximating zero. Only one patient (affected by psoriatic arthritis) experienced a disease flare and no cases of post-vaccination polymyalgic syndromes were observed, as reported in the literature [17]. Similarly, no relapses of HZ were observed in the general care group.

The RZV demonstrated a consistent favourable safety profile across both study cohorts, following administration of both doses. Among patients with rheumatologic diseases, AEFI were documented in 20 of 114 patients (17.5%) following the first dose and in 17 of 114 (14.9%) after the second (Table 2).

In the general care cohort, AEFI were reported in 14 of 65 individuals (21.5%) after the first dose and in 12 of 65 (18.5%) following the second administration. These AEFI were observed in patients receiving cDMARDs (n = 1; 0.9%), bDMARDs (n = 4; 3.5%), MMF (n = 1; 0.9%), JAK inhibitors (n = 6; 5.3%), a combination of cDMARDs and bDMARDs (n = 4; 3.5%) and a combination of cDMARDs and JAK inhibitors (n = 3; 2.6%). No statistically significant difference in the frequency of minor AEFI was observed between rheumatologic patients and general care (*p* = 0.326). AEFI were not significantly influenced by the number of attacks before RZV (*p* = 0.524), by comorbidities (*p* = 0.239) or by concomitant therapy with glucocorticosteroids (*p* = 0.264).

No significant association was found with disease duration and with advanced treatment including bDMARDs and/or JAK inhibitors or treatment with JAK inhibitors considered separately from other therapies, as detailed in Table 3.

## 4. Discussion

In our study, we observed a significant reduction in the VZV relapses before and after vaccine. In our cohort, only one case of HZ recurrence was observed 14 weeks after the second vaccine dose, aligning with the incidence trends in the general population. This finding was not influenced by concomitant immunosuppressive therapy overall, nor when JAK inhibitors were considered separately. Consequently, these data support maintaining immunosuppressive treatment during scheduled vaccination. The same tendency was observed for age, as no correlation was detected with the mean reduction on RZV relapses.

Patients with rheumatic diseases are known to be at increased risk for infections, including HZ, especially when receiving immunosuppressive therapy. This risk is particularly elevated in patients receiving JAK inhibitors [7], and could be influenced by treatment duration [18], the concomitant use of bDMARDs [2], corticosteroid exposure in a dose-dependent manner, longer disease duration and a prior history of HZ [19].

In line with these concerns, the 2019 update of EULAR recommendations for vaccination in adult patients with rheumatic diseases emphasizes the administration of the live-attenuated HZ vaccine at least four weeks before initiating bDMARDs or JAK inhibitors, but advises against its use during ongoing biologic therapy [8]. The RZV, licenced in Europe in 2018, demonstrating effectiveness and a safety profile in patients with pre-existing potential immune-mediated diseases comparable to that observed in the general population [14]. As a non-live vaccine, RZV thus emerged as a safer alternative to live-attenuated zoster vaccine (ZVL) for patients with rheumatic diseases. In this context, the 2022 American College of Rheumatology Guideline strongly recommended RZV for adult rheumatic musculoskeletal diseases (RMDs) patients receiving immunosuppressive therapy, despite the lack of direct evidence in this population, based on its proved immunogenicity and safety in patients undergoing renal transplantation [20] and with hematologic malignancies [21]. Although recent studies have addressed the immunogenicity, effectiveness, and safety of RZV in RMD patients, evidence is still limited. Nevertheless, studies specifically addressing RZV in RMD patients remain limited, and EULAR guidelines still lack clear definitions of “high-risk” patients and practical guidance on adjusting therapy at the time of vaccination [22].

Breakthrough cases reported in the literature include recurrences occurring 40 weeks [23,24] and 12 weeks [25,26] after the last RZV dose.

Since HZ frequently reactivates 7 to 44 weeks after the beginning of JAK inhibitors treatment, the former cases may have arisen as a complication related to the patients’ immunocompromised status. In the latter cases, the adjuvant in RZV may have triggered an inflammatory response, potentially leading to HZ reactivation. Furthermore, in the rheumatologic group, only one patient experienced a disease flare, characterized by worsening arthralgia, which required oral corticosteroids. This finding is consistent with the existing literature, which has demonstrated that the vaccine rarely impacts disease activity in well-controlled rheumatic patients undergoing bDMARDs therapy [27], although flares appear more frequent in RA [28].

In alignment with previous reports [16] and with findings in the general population, RZV demonstrated a favourable safety profile in our rheumatologic cohort. The most frequently reported AEFI were mild and transient, including fever and injection-site reactions, with no unexpected events observed. Notably, no cases of nausea or oral ulcers were reported. We did not observe significant correlations between the occurrence of minor AEFI and duration of the disease or with treatment with bDMARDs or targeted synthetic DMARDs (tsDMARDs) when considered separately. Belonging to the rheumatologic cohort rather than to the general care group does not predict the occurrence of adverse events. Consequently, the concomitant diagnosis of an immune-mediated disease does not present an impact on safety profile.

As Kluberg et al. reported no statistically significant association between the risk of incident gout during the 30-day risk period following RZV exposure compared to the control period [17], no cases of gout or gouty arthritis were observed in the months following RZV vaccination in our cohort. RZV is known to be highly reactogenic, with injection site reactions (4.39% in rheumatologic patients and 3.1% in the general care group) including redness, swelling and/or induration [29]. However, neither minor adverse events nor major adverse cardiovascular events were reported [30]. Although, Stefanizzi et al. [31] reported four cases of serious adverse events—including hyperpyrexia, anaphylaxis, and worsening dyspnea-in high-risk groups (immunosuppressed, oncologic, or dialysis patients)—none occurred in our cohort.

Emerging evidence indicates that the immunogenicity of the recombinant zoster vaccine (RZV) in patients with RMDs is largely comparable to that observed in the general population, although most investigations have been conducted in rheumatoid arthritis (RA) patients receiving JAK inhibitors. Data concerning other conditions, such as systemic lupus erythematosus (SLE), psoriatic arthritis (PsA), and ankylosing spondylitis (AS), remain limited [32]. Some studies have reported attenuated seroconversion rates and reduced antibody titers in RMD patients, findings that may be influenced by heterogeneous definitions of vaccine response [33]. While tofacitinib and baricitinib have been associated with modest impairments in pneumococcal vaccine responses [34,35], RZV immunogenicity in RA patients treated with JAK inhibitors appears preserved [25,29]. Evidence regarding upadacitinib is currently restricted to a single study [36]. By contrast, ZVL has demonstrated diminished immunogenicity in patients receiving bDMARDs, with response rates approximately half those of healthy controls [35]. However, in our study, antibody-mediated protection against HZ before and after vaccination could not be evaluated, as antibody titers were not measured.

Our study has several strengths, including the heterogeneous composition of the rheumatologic cohort with adequate representation of the main diagnostic categories. Importantly, RZV was administered to patients already receiving advanced therapies (bDMARDs and tsDMARDs), providing real-world data on effectiveness and safety in this population.

However, some limitations must be acknowledged: immunogenicity was not assessed through specific serum antibody titers which weakens the strength of the findings. Another limitation of this study is that exposures and outcomes, as well as their timing, were collected based on self-reported information, which introduces a recall bias thus potentially affected the accuracy of such information. Moreover, a limitation of our study lies in the very small number of HZ recurrences (only one case): although this finding may indicate that the vaccine is effective in preventing Zoster relapses, it could also weaken the validity of our conclusions. Furthermore, the optimal timing of vaccination in relation to DMARD initiation remains unclear, both in our study and in the literature.

In this regard, Takanashi et al. have designed a study to evaluate the timing of RZV administration in RA patients initiating tofacitinib, with anti-VZV IgG titers at week 12 as the primary endpoint [37]. The duration of vaccine-induced protection in immunocompromised populations is also uncertain and appears shorter than in the general population, with long-term real-life studies ongoing [38]. Finally, data regarding the observation time before vaccination in the general care group is lacking. Further research is required to clarify whether temporary interruption of immunosuppressive therapy could enhance vaccine effectiveness.

## 5. Conclusions

Our findings indicate that RZV is both effective and well tolerated in patients with rheumatic diseases, irrespective of diagnosis or ongoing therapy. The vaccine showed a reassuring safety profile, with no unexpected reactions. While additional studies are needed to better define immunogenicity and optimal timing in this population, our data strengthen the rationale behind the 2022 ACR recommendations and further support the broader use of RZV as a safe and valuable preventive strategy in patients with rheumatic disease undergoing immunosuppressive therapy.

## Figures and Tables

**Table 1:** Demographic and clinical characteristics of the cohort, assessed at the first vaccine dose.

Characteristics	Rheumatologic Patients	General Care
	Values, % (n)	Values, % (n)
**Total Number**	63.7% (114)	36.3% (65)
**Period of observation after first dose of vaccine (years)**	1.9 ± 0.93	-
**Period of follow up pre-RZV (years)**	5.69 ± 7.83	-
**Mean age ± SD (years)**	61.0 ± 14.3	77.0 ± 6.8
**Female (n, %)**	71.1% (81)	44.6% (29)
**Comorbidities (n, %)**	62.3% (71)	54.4% (62)
Previous malignancies	1.7% (2)	12.3% (8)
Other inflammatory/autoimmune disorder ^a^	18.4% (21)	21.5% (14)
Osteoporosis	16.7% (19)	0% (0)
Fibromyalgia	4.4% (5)	0% (0)
**Previous HZ, % (n)**	34.2% (39)	50.8% (33)
0, % (n)	65.8% (75)	49.2% (32)
1, % (n)	22.8% (26)	36.9% (24)
2, % (n)	7.9% (9)	10.8% (7)
3, % (n)	1.7% (2)	1.7% (2)
4, % (n)	1.7% (2)	0% (0)
**cDMARDs monotherapy, % (n)**	9.6% * (11)	-
MTX	5.3% * (6)	-
HCQ	2.6% * (3)	-
LFN	0.9% * (1)	-
APR	0.9% * (1)	-
**bDMARDs monotherapy, % (n)**		
ADA	8.8% * (10)	-
CZP	0.9% * (1)	-
ETN	3.5% * (4)	-
IFX	0.9% * (1)	-
IXE	0.9% * (1)	-
RTX	7.0% * (8)	-
SEC	1.7% * (2)	-
TCZ	9.6% * (11)	-
**JAKi monotherapy, % (n)**	28.1% * (32)	-
UPA	12.3% * (14)	-
BARI	11.4% * (13)	-
TOFA	3.5% * (4)	-
FILGO	0.9% * (1)	-
**Mycophenolate mofetil, % (n)**	7.9% * (9)	-
**bDMARDs + MTX, % (n)**	9.6% * (11)	-
**bDMARDs + other cDMARDs, % (n)**	3.5% * (4)	-
**JAKi+ MTX**	4.4% * (5)	-
**JAK i+ other cDMARDs, % (n)**	0.9% * (1)	-
**Oral GC**	28.1% * (32)	-

**List of abbreviations**: *ADA* adalimumab, *APR* apremilast, *BARI* baricitinib, *bDMARDs* biologic disease modifying anti-rheumatic drugs, *cDMARDs* conventional disease modifying anti-rheumatic drugs, *CZP* certolizumab, *ETN* etanercept, *FILGO* filgotinib, *GC* glucocorticoids, *HCQ* hydroxychloroquine, *HZ* herpes zoster, *IFX* infliximab, *IXE* ixekizumab, *JAKi* Janus Kinase inhibitors, *LFN* leflunomide, *MTX* methotrexate, *RTX* rituximab, *SEC* secukinumab, *TCZ* tocilizumab, *TOFA* tofacitinib, *UPA* upadacitinib. ^a^ Adrenal insufficiency, colitis, diabetes mellitus type 1, hyperthyroidism, hypothyroidism, latent autoimmune diabetes in adults, microscopic colitis, myasthenia gravis, pernicious anemia, primary biliary cirrhosis, psoriasis, pulmonary fibrosis, pyrophosphate arthritis, reactive arthritis, rosacea and urticaria. * frequency of use of a specific drug in that pharmacological class.

**Table 2:** Adverse event following immunization in rheumatologic and general care group.

AEFI *	First Dose				Second Dose			
	Patients		General Care Group		Patients		General Care Gorup	
	n	%	n	%	n	%	n	%
**Any reaction**	94	82	51	78.5	97	85.1	53	81.5
**General disorders**								
**Injection site reaction**	5	4.4	2	3.1	5	4.4	2	3.1
**Injection site rash**	0	0	2	3.1	0	0	0	0
**Fever**	10	8.8	8	12.3	7	6.1	8	12.3
**Flu-like syndrome**	1	0.9	0	0	2	1.8	0	0
**Nervous system disorders**								
**Headache**	1	0.9	0	0	1	0.9	0	0
**MSK and CTD**								
**Myalgia**	2	1.6	1	1.5	2	1.7	1	1.5
**Arthralgia**	1	0.9	1	1.5	0	0	1	1.5

**List of abbreviations**: CTD connective tissue disease; MSK musculoskeletal. * AEFI adverse events following immunization.

**Table 3:** Variables used in the binary logistic regression model alongside their respective *p*-values, odds ratios, and confidence intervals. AEFI is the dependent variable. All variables listed were included in a single multivariate model.

Variable	*p*-Value	Odd Ratio	C.I.
**Groups ^a^**	0.326	2.028	0.495–8.300
**Number of attacks before 1st dose of VZV vaccine ^b^**	0.524	0.780	0.362–1.677
**Comorbidities**	0.239	0.585	0.239–1.429
**Advance treatments ^c^**	0.311	1.941	0.510–7.380
**PDN or equivalent**	0.264	0.504	0.151–1.680

**List of abbreviations:** *C.I.* confidence interval, *PDN* prednisone, *VZV* varicella zoster virus. ^a^ Groups refer to rheumatologic vs. general care. ^b^ Number of attacks refer to prior flares. ^c^ Advance treatments include biologic and/or JAK-inhibitors.

## Data Availability

Data are available upon reasonable request to the corresponding author.

## Abbreviations

The following abbreviations are used in this manuscript:

AEFI	Adverse events following immunization
HZ	Herpes Zoster
RMDs	Rheumatic Diseases
RZV	Recombinant Zoster Vaccine
